# Social Support as a Key Protective Factor against Depression in HIV-Infected Patients: Report from large HIV clinics in Hanoi, Vietnam

**DOI:** 10.1038/s41598-017-15768-w

**Published:** 2017-11-14

**Authors:** Shoko Matsumoto, Kazue Yamaoka, Kenzo Takahashi, Junko Tanuma, Daisuke Mizushima, Cuong Duy Do, Dung Thi Nguyen, Hoai Dung Thi Nguyen, Kinh Van Nguyen, Shinichi Oka

**Affiliations:** 1 AIDS Clinical Center, National Center for Global Health and Medicine, Tokyo, Japan; 2 Graduate School of Public Health, Teikyo University, Tokyo, Japan; 3 Department of Infectious Diseases, Bach Mai Hospital, Hanoi, Vietnam; 4 National Hospital of Tropical Diseases, Hanoi, Vietnam

## Abstract

Depression is the most common mental health issue among people living with HIV/AIDS (PLWHA). This study explored how different types and sources of social support are associated with depression among HIV-infected patients in Vietnam. We carried out a cross-sectional survey on 1,503 HIV-infected patients receiving antiretroviral therapy at two HIV clinics in Hanoi in 2016. Depression was prevalent in 26.2% of participants. Higher score of social support, especially emotional/informational support and positive social interaction, showed significant association with lower depression rate. Although family was primary source of all types of social support, receiving emotional/informational support not only from family but also from outside of family correlated with a lower proportion of depression. In countries with constrained social resources and/or with family-oriented social structures, as in Vietnam, expanding social networks between HIV populations and society is a potentially important option for reducing depression.

## Introduction

The chronic nature of HIV has brought mental health issues to the fore as a critical problem in people living with HIV/AIDS (PLWHA). The most common mental health issue among PLWHA is depression, with the chance of developing a depressive disorder reportedly two to three times higher in PLWHA than in the general population^1,2^. Compared with PLWHA without depression, those with depression experience faster progression from HIV to AIDS^3,4^, higher mortality^3,5^, poorer adherence to antiretroviral therapy (ART)^6–8^, and greater prevalence of HIV risk behaviors^9–11^.

In Vietnam, a country with the fifth highest number of PLWHA in the Asia-Pacific region, the HIV epidemic is concentrated among high-risk groups (e.g., injection drug users [IDUs] and female sex workers)^12^, and many such people live with multiple stigma of HIV and drug use and/or sex work^13,14^. Additionally, depression in Vietnam is often recognized as a sign of immaturity or weak personality^15^. Amid this climate, the shortage of mental health professionals limits PLWHA in accessing mental health services^15^. Moreover, Vietnam is now facing withdrawal of international donors’ support for HIV-related services^16^, and the cost for ART will be allocated to the national health insurance scheme. This political change may impose substantial financial burden on patients, in the form of premiums and out-of-pocket costs. For these reasons, the HIV population in Vietnam is facing, or will be facing, greater risk of depression. However, few studies have addressed this issue in Vietnam^6,17–20^. These studies have had several limitations, such as relatively small sample size^18,19^, focusing on only a subgroup of the HIV population (e.g., males or sex workers)^18,20^, and using poorly validated instruments^17^.

There is a large body of evidence that social support plays a beneficial role in health^21–24^. Social support by definition is assistance people receive, or perceive, from their social networks. It is a multidimensional concept comprising *types* and *sources* ^25^. Major types of social support include emotional support (e.g., empathy, trust, or care), informational support (e.g., advice, suggestions, or information), tangible support (e.g., practical help, assistance, or financial support), and positive social interaction or social companionship (e.g., spending time with others in leisure and recreational activities)^26–28^. Sources of social support include family members, friends, neighbors, and colleagues. These two dimensions of social support may affect health and well-being in various ways^29–32^.

Compared with wealthy countries, low- and middle-income countries such as Vietnam have very limited social resources, such as support groups, available for HIV patients. Additionally, notably in Vietnam and often in other Asian countries, family is viewed as an extension of the self—a notion based on Confucian tradition—and family ties are much more interdependent and tightly knit than those in many Western countries^33,34^. Such countries may present no other options for HIV patients to seek support or assistance than through family when they are facing trouble. The perceptions and effectiveness of social support are closely interwoven into the socio-cultural context. Although a number of studies have shown a protective role of social support in depression in the HIV population^35–39^, we still do not have enough evidence on what social support available, and from whom, are most effective at protecting HIV patients against depression in countries with constrained social resources and/or with family-oriented cultures. Therefore, this study aimed to examine how different types and sources of social support are associated with depression among PLWHA in one such country: Vietnam.

## Methods

### Study design and study subjects

We conducted a self-administered questionnaire survey using a hospital-based cohort of PLWHA (aged ≥ 18 years). All participants were HIV-infected patients receiving ART. This “Hanoi cohort” was established in 2007 at two HIV outpatient clinics: National Hospital of Tropical Diseases (NHTD) and Bach Mai Hospital (BMH) in Hanoi. These are the largest referral clinics in Hanoi and are located next to each other.

A pilot survey was conducted for 2 days in December 2015, recruiting 67 patients who visited NHTD or BMH to verify the survey contents and test research procedures. We then conducted the actual survey during patients’ monthly visits between January and December 2016. We assigned a clinical staff member (nurse or social worker) to provide appropriate support for the participants. We excluded patients who participated in the pilot survey and who did not complete the questionnaire on depression from the present analyses.

### Measurements

#### Depression

Depression was evaluated using CES-D^33^. CES-D is a widely used self-reporting scale for measuring depressive symptoms that participants experience^34,35^. There is strong evidence for both its reliability and validation in Vietnam’s HIV population, with Cronbach’s alpha of 0.81, and sensitivity and specificity of 79.8% and 83.0%, respectively, at the cut-off score of 16^19^.

CES-D consists of 20 items. Responses were given on a four-point scale ranging from 0 (rarely or none of the time) to 3 (most or almost all the time), except for four items that were positively worded and scored in reverse. We used the Vietnamese version of CES-D, which was already available, and in this study defined a CES-D score of ≥16 as indicating depression. This cut-off score has been proven optimal for assessing depression in Vietnam’s HIV population^19^.

#### Social support

Types of social support were evaluated using the Medical Outcome Study Social Support Survey (MOS-SSS)^36^. This is a self-administered scale developed to measure perceived availability of types of social support, regardless of their sources. MOS-SSS consists of 19 items representing four types of social support; emotional/informational support (eight items), tangible support (four items), affectionate support (three items), positive social interaction (three items), and additional item (one item). The score for each item ranges from 0 (rarely or none of the time) to 5 (all of the time). MOS-SSS has been translated into various languages and adapted to different cultures and contexts^37–42^, however, because a Vietnamese version was not available, the English version was translated and then back-translated for use in this study to verify the accuracy of the translation. Additionally, in the pilot survey, respondents were asked to indicate any wording or expressions they did not understand or found unacceptable or offensive in view of cultural norms. After the pilot survey, an expert bilingual panel, including the original translator and health professionals, decided on the final version. Then, using the data from the main survey, Cronbach’s alpha was calculated to evaluate internal consistency, and confirmatory factor analysis was performed to examine the construct validity of Vietnamese version. Summed scores in each type of social support were used as continuous variables and categorical variables (quartiles) in the descriptive analyses, and as continuous variables in other analyses. Additional items were excluded from the analyses.

Information on sources of each type of social support in MOS-SSS was added to the questionnaire. Considering the prevalent family-oriented culture in Vietnam, we categorized sources of social support focusing on family and divided into the following groups: family only, family and others (i.e., partner, friends, medical staff, and others), lack of family (i.e., receiving support only from others), and none.

#### Demographics and HIV-related factors

The following data on demographic and HIV-related factors were collected: sex, age, IDU history, number of HIV-related symptoms, duration from HIV diagnosis, duration from ART initiation, history of EFV usage, latest CD4 count (/μl), and latest plasma viral load (pVL) (copies/ml). Age was divided into two categories: <35 years and ≥35 years. History of IDU was divided into three categories: current IDU (used injection drugs in the past 6 months), former IDU (history of injection drug use, but not used in the past 6 months), and non-IDU (never used injection drugs). HIV-related symptoms were measured using the 20-item HIV Symptom Index^43^. The total number of symptoms was used as a continuous variable. Duration from HIV diagnosis and from ART initiation were divided into the following categories: <1 year, 1–2 years, 3–4 years, and ≥5 years. History of EFV usage was divided into three categories: non-user, former user, and current user. CD4 count and pVL were tested in a laboratory, and data from the last clinic visit before the survey were obtained. These were divided into groups: <350 and ≥350, and <20 and ≥20.

#### Social factors

Information on social factors, other than social support, that was obtained included: residence, marital status, number of household members, educational attainment, employment, individual income, health insurance, and disclosure status. Residence was given as either Hanoi or others. Marital status was divided into four categories: not married, no partner; not married, but have a partner; married; and others (e.g., divorced or widowed). Number of household members was used as a continuous variable. Educational attainment was divided into three groups: low (never went to school, primary school, or junior high school), middle (high school), and high (vocational school/college or university). Employment was categorized as not employed, employed, or retired. Individual income was divided into the following categories: low (<1,500,000 Vietnamese dong [VND]), middle (1,500,000–4,999,999 VND), and high (≥5,000,000 VND). Health insurance and HIV disclosure status were evaluated dichotomously.

### Statistical analysis and ethics statement

We analyzed the prevalence of depression (CES-D ≥ 16) in accordance with the types and sources of social support the respondents perceived. Logistic regression analyses were then used to examine the statistical associations between social support and depression.

In the logistic regression analyses, we first focused on *types* of social support in relation to depression. In univariate models, we calculated the odds ratio (OR) with the 95% confidence interval (95%CI) for types of social support and other explanatory variables. A multivariate model was then developed to calculate the adjusted OR and 95%CI. Given the exploratory nature of the present analyses, we used a stepwise selection method for all variables (inclusion and exclusion criteria = 0.2 for each) in the multivariate models to improve statistical power. We employed four types of social support in the multivariate model to investigate which among them were predominantly associated with depression. In sensitivity analysis, considering the strong correlation between these four types, we developed four multivariate models in which each type was used individually to confirm the results of the prior model. Next, the *sources* of each type of social support as related to depression were further investigated. In this analysis, we used the sources of each social support type individually in the model.

By way of supplementary analyses, we calculated the adjusted OR and 95%CI of four variables—sex, age, IDU, and history of EFV usage—in every multivariate model by performing the stepwise selection method using forced entry of these variables. The variables were of interest because they had shown an association with depression in previous reports^15,20,44–48^.

All analyses were performed using SAS 9.4 software (SAS Institute Inc., Cary, NC, USA). All tests were two-sided, with the significance level set at 5%. Missing data were excluded from the analyses.

### Ethics statement

The study was approved by the Human Research Ethics Committee of the National Center for Global Health and Medicine (reference: NCGM-G-001845-00), BMH (reference: 19/BM-HDDD), and NHTD (reference: 12/HDDD-NDTU). We performed this study in accordance with the Japan Ethical Guidelines for Medical and Health Research Involving Human Subjects issued by Japan Ministry of Health, Labor and Welfare, in Dec 2014. Each participant provided written informed consent. Data were anonymized.

## Results

### Study participants

Since October 2007, 2,198 patients had registered for the Hanoi cohort, and 1,773 were still enrolled and underwent follow up in December 2015. Of those, 270 were excluded from the analyses: 134 who did not participate in the survey owing to their having no opportunity or time to take it, 67 from the pilot survey, and 69 who did not complete the CES-D. Thus, the present analysis included 1,503 patients.

Table 1 shows the respondents’ characteristics. The median age (interquartile range [IQR]) was 38 (33–42) years. In all, 22.2% of the respondents had a history of IDU. Among them, 1.5% were current users. Moreover, more than half of the respondents had been receiving ART for ≥5 years and 72.4% had been exposed to efavirenz (EFV).

**Table 1:** Characteristics of participants.

variables	n	%
**Demographics**		
**Gender**		
Male	903	60.08
Female	600	39.92
**Age**, median (IQR) years	38 (33, 42)	
<35 years	460	30.61
≥35 years	1043	69.39
**HIV-related factors**		
**History of IDU** ^a^		
Current IDU	23	1.53
Former IDU	311	20.69
Non IDU	1167	77.64
N/A	2	0.13
**Number of HIV-related symptoms**, median (IQR)	1 (0, 2)	
**Duration from HIV diagnosis**		
<1 year	11	0.73
1–2 year	134	8.92
3–4 year	331	22.02
≥5 years	1027	68.33
**Duration from ART initiation**		
<1 year	65	4.32
1–3 year	174	11.58
3–4 year	373	24.82
≥5 years	891	59.28
**History of EFV usage**		
Non-user	415	27.61
Former user	95	6.32
Current user	993	66.07
**Latest CD4 count**, median (IQR) (/µl)^b^	442 (322, 574)	
<350	437	29.08
≥350	1065	70.86
N/A	1	0.07
**Latest plasma viral load** (copies/ml)^b^		
<20	1261	83.9
≥20	240	15.97
N/A	2	0.13
**Social factors**		
**Residence**		
Hanoi	633	42.12
Others	866	57.62
N/A	4	0.27
**Marital Status**		
Not married and not having a partner	111	7.39
Not married but having a partner	48	3.19
Married	1296	86.23
Others (divorced, widow, etc.)	48	3.19
**Number of household members**, median (IQR)	4 (3, 4)	
**Educational attainment** ^c^		
Low	421	28.01
Middle	467	31.07
High	615	40.92
**Employment**		
Not employed	332	22.09
Employed	1093	72.72
Retired	69	4.59
N/A	9	0.60
**Individual income** ^d^		
Low	382	25.42
Middle	719	47.84
High	319	21.22
N/A	83	5.52
**Health insurance**		
No	280	18.63
Yes	1212	80.64
Unknown	11	0.73
**Disclosure status**		
No	69	4.59
Yes	1433	95.34
N/A	1	0.07

N/A: missing value.

^a^IDU: injection drug user; current IDU: used injection drugs in the past 6 months; former IDU: history of injection drug use, but not used in the past 6 months; non-IDU: never used injection drugs.

^b^Data at last clinic visit before the survey.

^c^Low: never went to school, primary school, or junior high school; Middle: high school; High: vocational school/college or university.

^d^Low: <1,500,000 Vietnamese dong (VND); Middle: 1,500,000–4,999,999 VND; High: ≥5,000,000 VND.

### Social support and depression

Table 2

**Table 2:** Prevalence of depression by type and source of social support.

Variables	n	%	CES-D ≥ 16 n (%)
All participants	1503	100.0	393 (26.15)
**Types of social support** ^**a**^			
**Emotional/Informational support (8–40)**, median (IQR)	30 (23, 35)		
Low (8–22)	314	20.89	146 (46.50)
Medium-low (23–29)	393	26.15	126 (32.06)
Medium-high (30–34)	364	24.22	83 (22.80)
High (35–40)	399	26.55	27 (6.77)
N/A	33	2.20	11 (33.33)
**Tangible support (4–20)**, median (IQR)	17 (14, 20)		
Low (4–13)	354	23.55	162 (45.76)
Medium-low (14–16)	303	20.16	88 (29.04)
Medium-high (17–19)	296	19.69	60 (20.27)
High (20)	537	35.73	78 (14.53)
N/A	13	0.86	5 (38.46)
**Affectionate support (3-15)**, median (IQR)	12 (9, 15)		
Low (3–8)	327	21.76	147 (44.95)
Medium-low (9–11)	270	17.96	84 (31.11)
Medium-high (12–14)	434	28.88	100 (23.04)
High (15)	456	30.34	56 (12.28)
N/A	16	1.06	6 (37.50)
**Positive social interaction (3-15), median (IQR)**	12 (9, 15)		
Low (3-8)	299	19.89	149 (49.83)
Medium-low (9–11)	374	24.88	121 (32.35)
Medium-high (12–14)	414	27.54	69 (16.67)
High (15)	410	27.28	52 (12.68)
N/A	6	0.40	2 (33.33)
**Sources of each social support type**			
**Source of emotional/informational support**			
Only family	603	40.12	187 (31.01)
Family and others^b^	735	48.90	145 (19.73)
Lack of family (only from others^b^)	143	9.51	53 (37.06)
None	19	1.26	6 (31.58)
N/A	3	0.20	2 (66.67)
**Source of tangible support**			
Only family	876	58.28	226 (25.8)
Family and others^b^	508	33.80	115 (22.64)
Lack of family (only from others^b^)	58	3.86	26 (44.83)
None	55	3.66	23 (41.82)
N/A	6	0.40	3 (50.00)
**Source of affectionate support**			
Only family	837	55.69	196 (23.42)
Family and others^b^	460	30.61	107 (23.26)
Lack of family (only from others^b^)	95	6.32	41 (43.16)
None	111	7.39	49 (44.14)
**Source of positive social interaction**			
Only family	724	48.17	174 (24.03)
Family and others^b^	525	34.93	118 (22.48)
Lack of family (only from others^b^)	179	11.91	66 (36.87)
None	73	4.86	35 (47.95)
N/A	2	0.13	0 (0.00)

N/A: missing value.

^a^categories were developed using quantiles of the score in each type of social support.

^b^others: partner, friends, medical staff, and others.

shows the prevalence of depression (CES-D ≥ 16) in accordance with the types and sources of social support the participants perceived. The overall prevalence of depression in this population was 26.2% (23.0% in men, 30.8% in women). A linear inverse association between the score of each social support type and depression was observed. Furthermore, more than 80% of patients were receiving each type of social support from their family. Only 30–40% of patients were receiving social support from someone outside their family, except for emotional/informational support (58.4%).

#### Types of social support and other factors

Types of social support were evaluated using the aforementioned Vietnamese version of MOS-SSS. In the main survey, this scale showed good internal consistency with Cronbach’s alpha, 0.95, for the overall score, and 0.90–0.93 for the four sub-scales. In confirmatory factor analysis, an original four-factor model of MOS-SSS provided the best-fitting structure, with a comparative fit index of 0.96 and adjusted goodness of fit index of 0.89. Construct validity of the Vietnamese version was thereby confirmed (Supplementary Figure S1).

Using this scale, we first investigated statistical association between four types of social support and depression. Table 3 shows the results from univariate and multivariate logistic regression models. In the multivariate model, sex, number of HIV-related symptoms, duration from HIV diagnosis, number of household members, employment, individual income, emotional/informational support, and positive social interaction were selected via a stepwise selection method and included in the final model. Among these variables, higher number of HIV-related symptoms (OR = 1.35, 95%CI: 1.25–1.46 per increase of one in number of symptoms), duration from HIV diagnosis (<1 year) (OR = 7.98, 95%CI: 1.48–43.11 vs. ≥ 5 years), and being unemployed (OR = 1.76, 95%CI: 1.18–2.61 vs. employed) were associated with higher proportion of having depression. However, high individual income (OR = 0.61, 95%CI: 0.38–0.98 vs. low individual income), and higher score for emotional/informational support and positive social interaction (OR = 0.94, 95%CI: 0.92–0.96, OR = 0.93, 95%CI: 0.89–0.98 per one-point increase in MOS-SSS score, respectively) were protectively associated with depression.

**Table 3:** Odds ratios and 95% confidence intervals of types of social support and other variables in univariate and multivariate logistic regression models.

variables	Univariate model		Multivariate model^a^ (n = 1,329)	
	OR (95%CI)	*p*	OR (95%CI)	*p*
**Sex**				
Male	1.00	<0.001	1.00	0.15
Female	1.49 (1.18–1.88)		1.24 (0.93–1.65)	
**Age (years)**				
<35	1.13 (0.88–1.45)	0.33	—	
≥35	1.00		—	
**History of IDU** ^**b**^				
Current IDU	2.60 (1.14–5.96)	0.07	—	
Former IDU	0.95 (0.71–1.27)		—	
Non IDU	1.00		—	
**Number of HIV-related symptoms**	1.41 (1.33–1.50)	<0.001	1.35 (1.25–1.46)	<0.001
**Duration from HIV diagnosis (years)**				
<1	2.55 (0.77–8.43)	0.13	7.98 (1.48–43.11)	0.03
1–2	1.12 (0.75–1.69)		1.35 (0.82–2.22)	
3–4	1.31 (0.99–1.72)		1.33 (0.95–1.87)	
≥5	1.00		1.00	
**Duration from ART initiation (years)**				
<1	1.00	0.37	—	
1–2	1.02 (0.53–1.94)		—	
3–4	1.18 (0.65–2.14)		—	
≥5	0.93 (0.52–1.64)		—	
**History of EFV usage**				
Non-user	1.00	0.09	—	
Former user	1.21 (0.75–1.95)		—	
Current user	0.81 (0.62–1.04)		—	
**Latest CD4 count** (/µl)^c^				
<350	1.00	0.34	—	
≥350	1.13 (0.88–1.46)		—	
**Latest plasma viral load** (copies/ml)^c^				
<20	1.00	0.25	—	
≥20	1.20 (0.88–1.62)		—	
**Residence**				
Hanoi	1.00	0.10	—	
Others	1.22 (0.96–1.54)		—	
**Marital Status**				
Not married, no partner	1.84 (1.23–2.76)	0.02	—	
Not married, but have a partner	1.38 (0.74–2.56)		—	
Married	1.00		—	
Others (divorced, widowed, etc.)	1.25 (0.66–2.35)		—	
**Number of household members**	0.76 (0.68–0.90)	<0.001	0.84 (0.70–1.00)	0.05
**Educational attainment** ^**d**^				
Low	1.66 (1.25–2.20)	<0.01	—	
Middle	1.33 (1.00–1.76)		—	
High	1.00		—	
**Employment**				
Not employed	2.41 (1.86–3.13)	<0.001	1.76 (1.18–2.61)	0.02
Employed	1.00		1.00	
Retired	0.98 (0.54–1.76)		0.98 (0.47–2.05)	
**Individual income** ^e^				
Low	1.00	<0.001	1.00	0.11
Middle	0.56 (0.43–0.73)		0.86 (0.59–1.24)	
High	0.31 (0.22–0.45)		0.61 (0.38–0.98)	
**Health insurance**				
No	1.14 (0.86–1.53)	0.37	—	
Yes	1.00		—	
**Disclosure status**				
No	1.08 (0.63–1.86)	0.78	—	
Yes	1.00		—	
**Emotional/Informational support**	0.91 (0.90–0.93)	<0.001	0.94 (0.92–0.96)	<0.001
**Tangible support**	0.89 (0.87–0.91)	<0.001	—	
**Affectionate support**	0.85 (0.83–0.88)	<0.001	—	
**Positive social interaction**	0.81 (0.79–0.84)	<0.001	0.93 (0.89–0.98)	0.01

OR: odds ratio; 95%CI: 95% confidence interval; *p*: p-value.

-: included in multivariate model, but not selected via stepwise selection methods for the final model.

^a^Stepwise selection method for all variables was used (inclusion and exclusion criteria = 0.2 for each).

^b^IDU: injection drug user, current IDU: used injection drugs in the past 6 months); former IDU: history of injection drug use, but not used in the past 6 months; non-IDU: never used injection drugs.

^c^Data at last clinic visit before the survey.

^d^Low: never went to school, primary school, or junior high school; Middle: high school; High: vocational school/college or university.

^e^low: <1,500,000 Vietnamese dong (VND); middle: 1,500,000–4,999,999 VND; high: ≥5,000,000 VND.

In the sensitivity analysis, wherein each type of social support was used individually in the multivariate model, the variables selected via the stepwise method were the same as those in the primary model, except that sex was not selected. In these models, all types of social support were significantly associated with lower proportion of having depression (OR = 0.92, 95%CI: 0.91–0.94 for emotional/informational support, OR = 0.91, 95%CI: 0.88–0.93 for tangible support, OR = 0.88, 95%CI: 0.85–0.91 for affectionate support, OR = 0.85, 95%CI: 0.82–0.88 for positive social interaction, per one-point increase in MOS-SSS score, respectively) (Table 4). The direction of effect and statistical significance of other explanatory variables in these models did not change from the primary model, except that the number of household members and duration from HIV diagnosis (3–4 years) showed statistical significance in all models.

**Table 4:** Sensitivity analyses: odds ratios and 95% confidence intervals of each type of social support individually used in four multivariate logistic regression models.

Type of social support	OR (95%CI)	*p*
Emotional/Informational support	0.92 (0.91–0.94)	<0.001
Tangible support	0.91 (0.88–0.93)	<0.001
Affectionate support	0.88 (0.85–0.91)	<0.001
Positive social interaction	0.85 (0.82–0.88)	<0.001

OR: odds ratio; 95%CI: 95% confidence interval; *p*: p-value.

Odds ratio was calculated in four multivariate models in which each type was used individually.

Odds ratios were adjusted by number of HIV-related symptoms, duration from HIV diagnosis, number of household members, employment, and individual income.

Except for types of social support, higher number of HIV-related symptoms, duration from HIV diagnosis (<1 year and 3–4 years vs. ≥5 years), and unemployment were associated with higher probability of depression; higher number of household members and high individual income were protectively associated with depression in all models.

#### Sources of social support

Next, we investigated the sources of each social support type for its relation with depression. Table 5 shows the results of univariate and multivariate logistic regression models. We used the sources of each social support type individually in four multivariate models. In these models, the variables selected via the stepwise method were the same as those in the primary model. With regard to tangible support, affectionate support, and positive social interaction, those not receiving such support from anyone (OR = 2.04, 95%CI: 1.07–3.87; OR = 2.29, 95%CI: 1.44–3.65; and OR = 2.15, 95%CI: 1.19–3.88, respectively) nor from their family (OR = 2.78, 95%CI: 1.49–5.18; OR = 3.05, 95%CI: 1.87–4.97; and OR = 1.76, 95%CI: 1.19–2.62, respectively), showed higher probability of having depression compared with those receiving such support from their family. However, the source of emotional/informational support showed a unique association with depression. Although not receiving emotional/informational support from one’s family was not significantly associated with depression, those receiving such support from both their family and others showed a lower rate of depression compared with those receiving it only from their family (OR = 0.58, 95%CI: 0.44–0.77).

**Table 5:** Odds ratio and 95% confidence intervals of sources of each social support type individually used in univariate and multivariate logistic regression models.

Source of support	Univariate model		Multivariate model	
	OR (95%CI)	*p*	OR (95%CI)	*p*
**Source of emotional/informational support**				
Only family	1.00	<0.001	1.00	<0.001
Family and others	0.55 (0.43–0.70)		0.58 (0.44–0.77)	
Lack of family (only from others)	1.31 (0.90–1.92)		1.08 (0.68–1.69)	
None	1.03 (0.38–2.74)		0.77 (0.23–2.55)	
**Source of tangible support**				
Only family	1.00	<0.001	1.00	<0.01
Family and others	0.84 (0.65–1.09)		0.93 (0.70–1.24)	
Lack of family (only from others)	2.34 (1.36–4.01)		2.78 (1.49–5.18)	
None	2.07 (1.19–3.61)		2.04 (1.07–3.87)	
**Source of affectionate support**				
Only family	1.00	<0.001	1.00	<0.001
Family and others	0.99 (0.76–1.30)		0.97 (0.71–1.32)	
Lack of family (only from others)	2.48 (1.61–3.84)		3.05 (1.87–4.97)	
None	2.59 (1.72–3.88)		2.29 (1.44–3.65)	
**Source of positive social interaction**				
Only family	1.00	<0.001	1.00	<0.001
Family and others	0.92 (0.70–1.20)		0.87 (0.64–1.18)	
Lack of family (only from others)	1.85 (1.30–2.62)		1.76 (1.19–2.62)	
None	2.91 (1.78–4.75)		2.15 (1.19–3.88)	

OR: odds ratio; 95%CI: 95% confidence interval; *p*: p-value.

Others: partner, friends, medical staff, and others.

Odds ratios was calculated when using each source of social support one by one in the multivariate models.

Odds ratios were adjusted by sex, number of HIV-related symptoms, and duration from HIV diagnosis, number of household members, employment, and individual income all model.

Except for sources of social support, higher number of HIV-related symptoms, duration from HIV diagnosis (<1 year and 3–4 years vs. ≥5 years), and unemployment were associated with depression; higher number of household members and high individual income were protectively associated with depression in all models.

#### Supplementary analysis

We calculated the adjusted OR and 95%CI for four variables—sex, age, IDU, EFV usage—in every multivariate model by applying the forced entry of these variables in the stepwise selection method. Although being female and being a current or former IDU were associated with higher rate of having depression in all the models, and being a current EFV user always showed a protective association with depression, these were not statistically significant. The direction of effect of age and being a former EFV user changed from model to model, and the association did not show statistical significance in any of the models.

## Discussion

The prevalence of depression was 26.2% in the present study sample. After controlling by various socio-demographic factors, social support was found to have a significant association with lower depression rate. Among four types of social support, emotional/informational support and positive social interaction were found predominantly associated with a lower proportion of people with depression. Although family was the primary source of all types of social support, those receiving emotional/informational support not only from family but also from people outside that family showed a lower proportion of having depression.

The prevalence of depression found in this study (26.2%) was relatively lower than that in previous studies targeting Vietnam’s HIV population, and using the same cut-off score for CES-D (36.5–78.0%)^6,18,19^. However, the prevalence in the present study was near that reported for Vietnam’s general population (24.3%)^49^. This could be because we included patients in a stabilized HIV condition (e.g., 71% with CD4 ≥ 350) and who were receiving HIV treatment in tertiary-level facilities. Our result may be a hopeful sign suggesting engagement in continuous HIV treatment and care with better quantity and quality of resources could help patients maintain a healthy mental status at rates comparable to those of the general population.

Social support was found significantly associated with a lower depression rate. This association was stronger than that with previously reported risk factors of depression (e.g., sex, age, IDU, EFV usage), and did not change even after controlling by various socio-demographic factors. Although all four types of social support examined individually showed a protective association with depression, this association was most prominent between emotional/informational support and positive social interaction, and depression. This is somewhat consistent with previous findings, which suggested social support, especially emotional support, buffers the deleterious influences of stressful events and mitigates the risk of depression^50–53^. The chronic course of HIV infection requires patients to cope with various forms of psychosocial stress associated with opportunistic infection, side effects of ART, and social prejudice and discrimination^54^. Therefore, our findings may suggest that, even though most participants had been receiving ART for a long period and their health status was stable, those without social support tended to be facing substantial psychological stress and were at a greater risk of contracting depression. For such a population, compared with practical support, emotional/informational support and positive social interaction may be more effective support forms for mitigating their stress, and thereby safeguarding against depression. Additionally, the importance of positive social interaction found in this study may reflect a limited social network between HIV populations and general society. In Vietnam, drug users and sex workers have been critically labeled as “social evils” in relation to HIV transmission^15^. Social discrimination and prejudice against the HIV population resulted in discouraging such people from disclosing their HIV status to anyone outside their family^15,55^. Indeed, in our study, while more than 90% of patients had disclosed their status to their family, only 14% had done so to their friends.

As previously reported, we also found family to be the vital source of all types of social support among HIV patients; we found participants who lacked tangible support, affectionate support, and positive social interaction from their family had a higher probability of having depression. Interestingly, however, regarding emotional/informational support, lack of family support itself was not associated with depression. Rather, receiving such support not only from family, but also from people outside the family, was protectively associated with depression. This result may have important implications for social support intervention for mental health. Although it has been well-known that forms of family-based intervention, such as involvement of family in HIV therapy or provision of educational programs for family, could help foster patients’ mental health^56–58^, people outside the family may be also important contributors, even in family-oriented societies. With such social structures as in Vietnam, those without family support may face difficulty and stress in disclosing their HIV status to their family and seeking support from them. Considering that close relationships represented by family ties may be a potential source of stress, and may have a deleterious influence on health^59–61^, programs aimed at bolstering social support by expanding social networks to people outside the family, rather than by strengthening family support, may be more effective at fostering mental health in such cases.

Notably, our study contained a unique system for HIV treatment and care, in which treatment groups were organized. Professionals formed groups of 10–30 previously unacquainted HIV-infected patients who started ART in the same month. Subsequently, medical follow-up schedules, adherence counseling sessions, and other activities were arranged. This system may naturally create social ties between patients, and foster peer support among them. It may also have contributed to the lower prevalence of depression found in this study, and be a potential and already existing resource to strengthen social support.

Our study showed low prevalence of depression among HIV patients engaged in continuous HIV treatment and care with appropriate resources. However, considering the association of unemployment and low individual income with depression found in this study, the shifting financial burden for HIV services may exert further psychological burden on patients, and increase their risk of depression. Against this backdrop, this is the first investigation on two dimensions of social support (types and sources) and depression in Vietnam, using a large sample of the HIV-infected population. We found potential effectiveness of social support as an alleviator of depression, and recognized the importance of people outside the family as a source of such support. The findings of our study could provide useful information for future mental health strategies in other resource-constrained settings and in family-oriented societies. However, there are several limitations in this study. First, we adopted a cross-sectional design, which limits causal inferences for the associations found. The association between lack of social support and depression found herein may reflect reverse causation (i.e., people with depression do not seek social support). Second, our study sites were two large referral HIV clinics located in a main city; therefore, the characteristics of the participants may not accurately represent Vietnam’s entire HIV population. Third, CES-D is not a diagnostic instrument. Our findings can only be interpreted in association with depressive symptomatology. Fourth, with the forward- and back-translation processes and the results of Cronbach’s alpha and confirmatory factor analysis, the content and the construct validity of the Vietnamese version of MOS-SSS were reinforced to some extent, but the scale’s cultural adaptation and validity should be further explored using more standardized methodologies including the guidance developed by the Translation and Cultural Adaptation working group in the International Society for Pharmacoeconomics and Outcomes Research^62^. Finally, we did not assess supporters’ relationships and dynamics in relation to depression. Such information is needed for formulating a strategic plan to address depression.

In conclusion, the prevalence of depression found in this study was low compared with that in previous reports. Even in countries with constrained social resources and/or with family-oriented culture, people outside the family are potentially important in promoting mental health in HIV-infected patients. It is important to expand social networks between HIV populations and general society, especially for those with less family support.

### Data availability

The datasets generated during and/or analyzed during the current study are available from the corresponding author on reasonable request.

## Electronic supplementary material

Supplementary Figure S1
