# Supplementary material for: Social Support as a Key Protective Factor against Depression in HIV-Infected Patients: Report from large HIV clinics in Hanoi, Vietnam
Source: Sci Rep. 2017 Nov 14;7:15489. doi: 10.1038/s41598-017-15768-w (PMC5686163; doi:10.1038/s41598-017-15768-w)
Supplement: Supplementary file 1 — Supplementary Figure S1 [file 41598_2017_15768_MOESM1_ESM.pdf]

Social Support as a Key Protective Factor against Depression in HIV-Infected Patients: Report from large HIV clinics in Hanoi, Vietnam

Shoko Matsumoto, Kazue Yamaoka, Kenzo Takahashi, Junko Tanuma, Daisuke Mizushima, Cuong Duy Do, Dung Thi Nguyen, Hoai Dung Thi Nguyen, Kinh Van Nguyen, Shinichi Oka

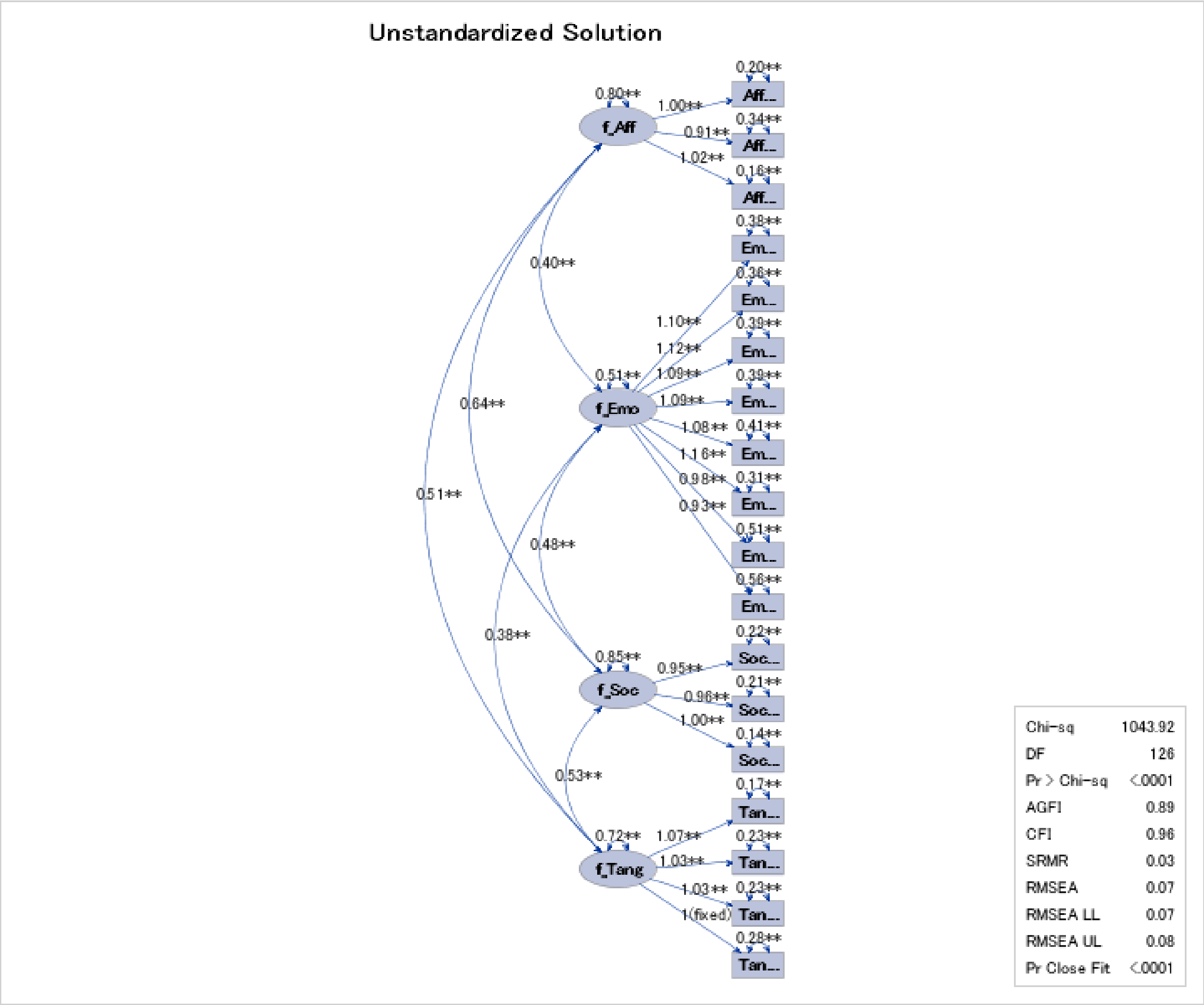

Supplementary Figure S1. Path diagram of the confirmatory factor analysis of the Vietnamese version of MOS-SSS
